# HLA Alleles Associated with Slow Progression to AIDS Truly Prefer to Present HIV-1 p24

**DOI:** 10.1371/journal.pone.0000920

**Published:** 2007-09-19

**Authors:** José A. M. Borghans, Anne Mølgaard, Rob J. de Boer, Can Keşmir

**Affiliations:** 1 Theoretical Biology/Bioinformatics, Utrecht University, Utrecht, The Netherlands; 2 Department of Immunology, University Medical Center Utrecht, Utrecht, The Netherlands; 3 Center for Biological Sequence Analysis, Technical University of Denmark, Lyngby, Denmark; 4 Academic Biomedical Centre, Utrecht University, Utrecht, The Netherlands; Massachusetts General Hospital, United States of America

## Abstract

**Background:**

The mechanism behind the association between human leukocyte antigen (HLA) molecules and the rate of HIV-1 disease progression is still poorly understood. Recent data suggest that “protective” HLA molecules, i.e. those associated with a low HIV-1 viral load and relatively slow disease progression, tend to present epitopes from the Gag capsid protein. Although this suggests that preferential targeting of Gag delays disease progression, the apparent preference for Gag could also be a side-effect of the relatively high immunogenicity of the protein.

**Methods and Findings:**

To separate cause and effect, we predicted HIV-1 epitopes from the whole genome of HIV-1, and found that protective HLA alleles have a true preference for the p24 Gag protein, while non-protective HLA alleles preferentially target HIV-1 Nef. In line with this, we found a significant negative correlation between the predicted affinity of the best-binding p24 epitopes and the relative hazard of HIV-1 disease progression for a large number of HLA molecules. When the epitopes targeted by protective HLA alleles were mapped to the known p24 structure, we found that mutations in these epitopes are likely to disturb the p24 dimer structure, which is expected to severely reduce the fitness of the virus.

**Conclusions:**

Our results suggest that the intrinsic preference of different HLA molecules to present p24 peptides explains why some HLA molecules are more protective than others.

## Introduction

There is increasing evidence that human leukocyte antigen (HLA) molecules influence the rate of disease progression after HIV-1 infection (reviewed in [1]). Cytotoxic T lymphocyte (CTL) responses against HIV-1 assert a strong evolutionary pressure on the virus, causing selection of HIV-1 variants that successfully escape recognition by CTL [2]–[4]. Due to the large polymorphism of HLA molecules, different individuals are able to respond to specific immunodominant HIV-1 epitopes depending on their HLA background. Since disease progression is influenced by an individual's HLA background, some CTL responses apparently control the virus better than others.

HLA-B57 molecules have been found to have the strongest association with immune control of HIV-1 (see e.g. [5], [6]). Among elite suppressors of HIV, i.e. HIV-infected individuals with normal CD4^+^ T cell counts and viral loads below the detection level without therapy, HLA-B57 molecules are significantly overrepresented [7]. HLA-B57 restricted CTL responses tend to be more responsive and immunodominant than other CTL responses [8]–[10]. HLA-B58 and HLA-B63 have binding motifs that are very similar to HLA-B57 and are also associated with good immune control of HIV-1 [1], [11]. The protective effect of HLA-B27, another HLA type associated with long term non-progression [1], has been proposed to be due to the very conserved Gag epitope it presents [12]. HLA-B35 and HLA-B53, on the other hand, tend to be associated with relatively rapid progression to AIDS [1], although different associations have been found for different HLA-subtypes: while HLA-B3503 is associated with rapid progression to AIDS, the rate of disease progression of individuals with HLA-B3501 (which has only a slightly different peptide-binding motif) does not differ from the population average [13].

The frequency of HLA molecules in the human population has been proposed to play a role in the association between HLA molecules and the rate of disease progression. Firstly, individuals with rare HLA molecules are more likely to be heterozygous at the HLA loci, and are thereby expected to induce an immune response against a larger diversity of peptides than homozygous individuals. Indeed, HLA-heterozygosity is associated with relatively slow disease progression in HIV-1 infection [13], [14]. Secondly, since HIV-1 evolution occurs in the context of the HLA background of the human population, HIV-1 may be better adapted to common than to rare HLA molecules [15]–[17]. However, a large study establishing the relative hazard of HIV-1 disease progression for different HLA molecules found no correlation between the relative hazard and the population frequency of HLA molecules [13].

Alternatively, qualitative differences between HLA molecules in terms of the specific HIV peptides that they present may explain the association between HLA molecules and the rate of HIV disease progression. It has recently become clear that individuals with slow HIV-1 disease progression tend to make broad and strong CTL responses against HIV-1 Gag, while individuals with rapid disease progression and high HIV-1 viral loads make strong CTL responses to Env and accessory/regulatory proteins [7], [18]–[25]. It is tempting to conclude from these data that certain HLA alleles provide better protection against HIV-1 disease progression because of their tendency to induce CTL responses to HIV-1 Gag. The observed association between the presence of CTL to Gag and slow disease progression could, however, also be due to the relatively high immunogenicity of HIV-Gag [26], which would give a bias towards the detection of Gag-specific responses in individuals with low viral loads. In the latter case, the observed association between HLA molecules with a low relative hazard and CTL responses against Gag would not be the cause but the consequence of low viral loads.

To separate cause and effect, we studied the binding preferences of different HLA alleles for the different HIV proteins using HLA-peptide prediction tools. The use of prediction tools, instead of clinical data, is crucial in this analysis, because prediction tools will reveal unbiased preferences of HLA alleles for different HIV-1 proteins, whereas HLA-restricted HIV-1 epitopes reported in the HIV-1 database may be biased, because some HIV-1 proteins are studied more extensively than others. We found that HLA molecules associated with slow HIV disease progression have an intrinsic preference to present epitopes from the p24 Gag capsid protein of HIV-1. Analysis of the structure of the p24 protein pointed out that CTL escape mutations restricted by protective HLA molecules are likely to disturb the ability of p24 to form a dimer and thereby to result in a drastic viral fitness loss. Taken together, our analyses suggest that differences between HLA molecules in the tendency to present peptides from p24 underlie the association between HLA molecules and the rate of HIV-1 disease progression.

## Methods

### Data

The consensus and ancestral sequences for different HIV proteins, as well as 59 individual world-wide HIV-1 clade B p24 sequences with known year of sampling were downloaded from the Los Alamos HIV database at www.hiv.lanl.gov/ (consensus August 2004).

### HLA-peptide binding predictions

We used two different algorithms to predict the binding affinities of HIV-1 peptides (of length 9 and 10) to HLA molecules: the stabilized matrix method (SMM), available at www.immuneepitope.org [27], and an artificial neural network (ANN), NetMHC3.0 available at www.cbs.dtu.dk/services/NetMHC-3.0/ [28], [29]. For HLA-B3503 we used the only method that is currently available, i.e. the general matrix method at www.cbs.dtu.dk/services/NetMHC-3.0/. Since prediction scores from these algorithms for different HLA molecules cannot be compared directly, we either used the ranks of these peptides among all HIV-1 peptides, or applied an HLA-binding affinity normalization as previously proposed [30]. Briefly, the HLA-binding affinities of HIV-1 peptides were divided by an HLA-specific threshold, which was based on the top 1% binding affinities of a large set of non-HIV peptides.

### Sequence logos

Sequence logos [31] were made to visualize i) to what extent a position in a sequence is conserved (given by the height of a bar, i.e. the information content) and ii) which amino acids are most frequently found at a particular position (the height of each amino acid in the logo is proportional to the frequency of occurrence at that position). Sequence logos were generated using the Shannon information content [32]. The maximum information content is log _2_ 20 = 4.3, which is obtained if the same amino acid is always observed at a particular position. The minimum Shannon information is zero, which is obtained if all amino acids occur at the same frequency at a position.

## Results

### Protective HLA alleles preferentially target the p24 protein

**Figure 1:**
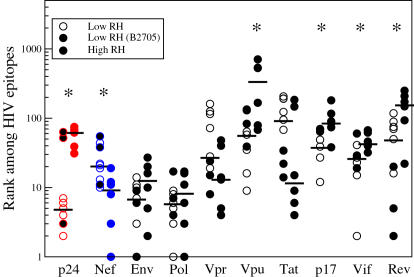
Predicted ranks of epitopes from different HIV-1 proteins. HLA-binding epitopes were predicted for HLA alleles with a low RH (HLA-B5701, HLA-B5801 and HLA-B2705) and HLA alleles with a high RH (HLA-B3503 and B5301), and their rank among all other HIV-1 epitopes was plotted. Low ranks correspond to good binding peptides. The analyses were limited to the three best-binding epitopes from each HIV-1 protein. HLA alleles with a low RH were found to have a significantly higher preference for peptides from p24 than HLA alleles with a high RH (*p* = 0.01, Mann-Whitney test), while HLA alleles with a high RH had a significantly higher preference for Nef (*p* = 0.02). A significant difference between low and high RH HLA molecules was also observed for peptides from Vpu (*p* = 0.01), p17 (*p* = 0.02), Vif (*p* = 0.02), and Rev (*p* = 0.01), but the median ranks of the best-binding peptides from these proteins are so high that these differences are probably not physiologically important. Epitopes for HLA-B2705 are depicted as shaded circles. All predictions were based on a neural-network based predictor (NetMHC); the use of matrix methods gave similar results (results not shown). The number of amino acids in each of the HIV proteins is: p24 231, Nef 206, Env 856, Pol 947, Vpr 96, Vpu 82, Tat 101, p17 132, Vif 192, and Rev 116 amino acids.

To investigate whether the recently reported association between HLA molecules conferring slow HIV-1 disease progression and CTL responses against Gag [7], [18]–[24] is due to intrinsic properties of HLA molecules or is a side-effect of the high immunogenicity of Gag, we analyzed HLA-peptide binding predictions for a large number of different HLA molecules. We studied the HLA-binding affinities of HIV-1 peptides for HLA-B5701, B5801, and B2705, three HLA alleles with a clearly low relative hazard (RH) of HIV disease progression [1] (Figure 1, Low RH, open and shaded symbols), and for HLA-B3503 and B5301, which are both associated with relatively rapid HIV-1 disease progression [1] (Figure 1, High RH, solid symbols). Predictions were made for all peptides of length nine and ten from the consensus sequence of HIV-1 clade B, using the stabilized matrix method [27], and an artificial neural network [28], [29] (see Methods). The HIV-1 epitopes were subsequently ranked by their predicted HLA-binding affinity within all HIV proteins. We plotted the ranks of the three best-binding peptides from each HIV-1 protein among all other HIV epitopes, because Kiepiela *et al.* [23] reported that the presence of CTL responses against at least two Gag-epitopes is associated with a low HIV-1 viral load.

Figure 1 shows that the three HLA alleles with a low RH have a significantly stronger preference for peptides from p24 than the two HLA alleles with a high RH (Mann-Whitney, *p* = 0.01), while the latter have a stronger preference for peptides from Nef (Mann-Whitney, *p* = 0.02). These differences remain significant when correcting for multiple tests of significance using the improved Bonferroni procedure [33]. It is also evident from Figure 1 that, despite the relatively small size of the p24 protein (7.5% of the HIV-1 proteome), many of the best-binding peptides for HLA alleles with a low RH come from p24. For HLA molecules with a low RH, the average rank of epitopes from p24 is comparable to the epitopes from the much larger proteins Env and Pol. The other relatively small HIV-1 proteins (p17, Vpr, Vpu, Tat, Vif and Rev) contain hardly any of the high ranking epitopes. Of note, both HLA-B5701 and B5801 appeared to have three very good-binding p24 epitopes, all ranking within the first ten among all HIV peptides. In contrast, HLA-B2705 was found to have only a single good-binding p24 epitope (see Figure 1, shaded symbols).

**Figure 2:**
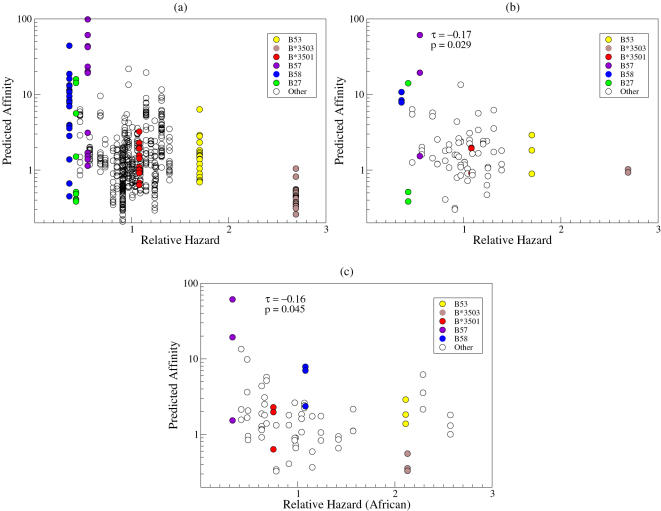
Relationship between relative hazard and predicted HLA-binding affinity for p24 epitopes. The relationship between the relative hazard of HIV-1 disease progression and the predicted affinity of p24 peptides plotted for different HLA alleles. The graphs show the normalized predicted affinity of the three best-binding p24 epitopes for each HLA allele. Data were based on (A) the Caucasian RH of 27 different HLA alleles [13] and 59 individual HIV-1 clade B sequences downloaded from the Los Alamos database, (B) the Caucasian RH of 27 different HLA alleles [13] and the consensus HIV-1 clade B sequence, and (C) the African RH of 24 different HLA alleles for which non-trivial RH have been determined [13] and the consensus HIV-1 clade C sequence. The RH values represent the relative hazard of the 2-digit HLA serotype (except for HLA-B3503 and B3501 for which separate RH are known [13]), while the predicted affinities were based on prediction tools for the 4-digit HLA allele that dominates the 2-digit HLA serotype (except for HLA-A0205 and HLA-B5802, which dominate the HLA serotypes HLA-A02 and HLA-B58 in Africans, but for which no high quality prediction tools are available; when they were omitted from the analysis in panel (C), the correlation remained significant (*p* = 0.014)). Because binding motifs of HLA molecules are not always independent, we repeated the analysis by randomly selecting only one peptide, when a peptide binds more than one allele. In all cases the analysis for HIV-1 clade B and clade C remained significant (*p*<0.02). All HLA-peptide binding predictions were based on matrix methods; very similar results were obtained when using NetMHC, or when we confined our analyses to the best-binding p24 peptide for each HLA type.

For a large number of HLA molecules, we plotted the predicted affinity score of the three best-binding p24 epitopes as a function of the relative hazard of the corresponding HLA type as defined by Gao *et al.* [13] (see Figure 2). It was previously established that these relative hazards do not significantly correlate with the frequencies of the HLA molecules in the human population (Kendall's *τ* = −0.08, *p* = 0.52, [13]). In contrast, we found a clear correlation between the affinity score of the best-binding p24 epitopes from 59 different individual HIV-1 clade B sequences and the RH of HIV-1 disease progression for 27 different HLA types (Figure 2A). Since the data from different HIV-1 clade B sequences are not independent, we used the consensus HIV-1 clade B sequence to test the significance of this correlation. This revealed a significant correlation between the binding affinity of epitopes from HIV-1 clade B p24 and the risk of HIV-1 disease progression (Kendall's *τ* = −0.17, *p* = 0.029, see Figure 2B). The best-binding HIV-1 clade B p24 peptides restricted by the protective HLA types B58 and B57 turned out to have significantly higher predicted affinity scores (purple and blue circles in Figure 2B) than those restricted by all other HLA alleles (Mann-Whitney, *p* = 0.0009). For none of the other HIV-1 clade B proteins, a significant correlation between the affinity score of the best-binding peptides and the relative hazard of disease progression of the different HLA types was found (not shown). Obviously, the HLA binding affinity does not have to correlate directly with immunogenicity or antiviral CTL response. However, as pointed out recently [34], HLA-binding is the most restrictive step in determining immunodominance. Taken together, these results suggest that the experimentally observed association between protective HLA alleles and responses against Gag is caused by an intrinsic preference of these HLA alleles for peptides from p24, and is not merely a side-effect of the high immunogenicity of Gag. The antigen processing efficacy (here measured as a combination of proteasome cleavage predictions [35] and predicted TAP binding affinity [36]) does not influence this intrinsic preference (results not shown).

If HIV-1 were adapting to the most common HLA alleles in the human population, one could argue that the current HIV-1 consensus sequence in the Caucasian population should contain more CTL escape mutations for common HLA types, such as HLA-B35, than for rare HLA types, such as HLA-B57, B58 and B27. We therefore repeated our analysis using the predicted ancestral sequence for HIV-1 subtype B and the HXB2 strain dating from 1983 (see Methods and [37]), which should contain no or only few CTL escape mutations. Both the HXB2 and the ancestral sequence differed by only 2 amino acids from the consensus HIV-1 clade B sequence; as a consequence there was hardly any difference between the predicted p24 epitopes from the consensus and the ancestral HIV-1 clade B sequences, and HXB2 for the various HLA types (results not shown). Additionally, the binding affinities of p24 epitopes from the consensus sequence of HIV-1 clade C, the major HIV-1 subtype in large parts of Africa, correlated significantly with the relative hazard of these HLA alleles in the Afro-American population [13] (Figure 2C). These observations demonstrate that our results are not due to frequency-dependent adaptation of HIV-1 to the most common HLA alleles in the human population, and thereby confirm that HLA types with a low RH have an intrinsic preference for peptides from p24.

### Preferential binding of p24 is due to sequence patterns

**Figure 3:**
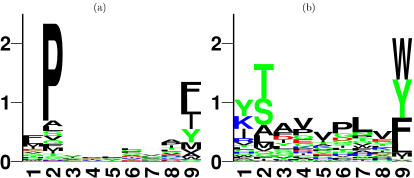
Binding motifs of HLA alleles with a low or high RH. The sequence logos displaying the preferences of HLA alleles with a low (A) or high (B) RH of HIV disease progression. Experimentally verified good-binding peptides for HLA-B5701 and B5801 were used to make the low RH logo (A), while the good binders of HLA-B3501 and B5301 were used for the high RH logo (B, data from www.immuneepitope.org). These sequence logos visualize i) to what extent a position in a sequence is conserved (given by the height of a bar, i.e. the information content) and ii) which amino acids are most frequently found at a particular position (the height of each amino acid in the logo is proportional to the frequency of occurrence at that position). The information content may vary between log _2_ 20 = 4.3, which is obtained if the same amino acid is always observed at a particular position, and zero, which is obtained if all amino acids occur at the same frequency at a position. Amino acids are colour coded according to their physicochemical characteristics. Neutral and polar, green; basic, blue; acidic, red; neutral and hydrophobic, black.

The differential targeting of p24 and Nef by protective and non-protective HLA alleles could be due to differences in the amino acid distributions or in the sequence patterns between the two proteins. When the amino acid residues occurring in p24 and Nef were completely shuffled (without changing the amino acid frequencies of the two proteins), both proteins were no longer preferentially targeted by any of the HLA alleles (results not shown). This suggests that p24 and Nef carry distinct sequence patterns that are preferred by HLA alleles with a low or high RH, respectively. To study the sequence patterns preferred by protective and non-protective HLA alleles, we devised sequence logos for both groups of HLA alleles, based on a large set of experimentally verified good-binding peptides (data available at www.immuneepitope.org). Since HLA-B57 and B58, and HLA-B35 and B53 are known to have similar binding motifs [38] we generated combined binding logos for the HLA alleles with low and high RH (see Figure 3). The sequence logos point out that the two groups of alleles have non-overlapping preferences, and target very different HIV-1 peptides. Taken together, the HLA-peptide binding data suggest that different sequence patterns occurring in HIV-1 p24 and Nef are causing the preferential binding of p24 and Nef peptides by HLA alleles associated with slow and rapid disease progression, respectively.

### Predicted associations with slow progression

Based on the suggestion that CTL responses directed against p24 slow down disease progression, we investigated which other HLA molecules and CTL epitopes are likely to be associated with slow disease progression, by predicting the p24 epitopes (based on consensus HIV-1 clade B) for 19 different (4-digit) HLA-A and 16 HLA-B alleles. The p24 peptides that are predicted to be among the 10 best-binding HIV peptides for each HLA molecule, which are all expected to be very good HLA binders, are summarized in Table 1. None of the HLA alleles with a high RH had a p24 epitope with a rank lower than 20. One forth of all epitopes in Table 1 are presented by HLA alleles with a binding motif very similar to HLA-B58 (belonging to the B58 supertype [38]). Interestingly, none of the good-binding p24 peptides came from HLA-B5802, while 4 good-binding p24 peptides came from HLA-B5801, which is fully in line with the recent observation that HLA-B5801 is associated with slow disease progression, while B-5802 is not [23]. We predict HLA-A6901 to be a protective HLA allele, having three very good, possibly immunodominant, p24 epitopes (see Table 1). This relatively rare HLA allele is not widely reported to be associated with slow HIV disease progression, even though HLA-A6901 has a RH as low as 0.47 [13], which is even lower than the RH of HLA-B57. Some of the other alleles in Table 1, including A0301, A2402, A2403, B0801 and B4501, have been reported to have a low RH among African-Americans [13]. In principle, all 18 HLA alleles and all CTL epitopes listed in Table 1 are predicted to be associated with slow disease progression. Thirty percent of these CTL epitopes remain to be experimentally confirmed.

**Table 1:** Predicted p24 epitopes with a high ranking^a^ HLA-binding affinity score.

*Epitope* b	*Position* c	*HLA* d	*Rank* e		*Supertype*		*Confirmation* j
			SMM^f^	ANN^g^	[38] h	[59] i	
FRDYVDRFY	161	A0101	>10	9	A1s	A1s	
HQAAMQMLK	60	A0301	3	>10	A3s	A3s	
RDYVDRFYK	162	A1101	>10	4	A3s	A3s	
GWMTNNPPIP	116	A2402	8	–	A24s	A24s	
STLQEQIGW	109	A2403	8	>10	A24s	A24s	
EVIPMFSAL	35	A2601	8	5	A1s	A26s	++
RDYVDRFYK	162	A3001	7	>10	A24s	A1s	.
RDYVDRFYK	162	A3101	>10	2	A3s	A3s	.
EVIPMFSAL	35	A6802	2	–	A2s	A2s	
MTNNPPIPV	118	A6802	6	–	A2s	A2s	.
EVIPMFSAL	35	A6901	>10	1	A2s	A2s	
EMMTACQGV	118	A6901	1	8	A2s	A2s	.
MTNNPPIPV	213	A6901	2	>10	A2s	A2s	.
DCKTILKAL	197	B0801	9	2	–	–	++
EIYKRWIIL	128	B0801	>10	8	–	–	++
KRWIILGLNK	130	B2705	3	3	B27s	B27s	++
GEIYKRWII	127	B4001	–	6	B44s	B44s	
REPRGSDIA	97	B4002	8	–	B44s	B44s	
SEGATPQDL	44	B4403	8	–	B44s	B44s	++
REPRGSDIA	97	B4501	2	–	B44s	B44s	
AEWDRLHPV	78	B4501	3	–	B44s	B44s	.
HPVHAGPIA	84	B5401	3	–	B7s	B7s	.
ISPRTLNAW	15	B5701	2	–	B58s	B58s	++
STLQEQIGW	109	B5701	4	–	B58s	B58s	++
QASQEVKNW	176	B5701	6	–	B58s	B58s	++
STLQEQIGW	109	B5801	2	>10	B58s	B58s	++
QASQEVKNW	176	B5801	4	>10	B58s	B58s	++
ISPRTLNAW	15	B5801	>10	6	B58s	B58s	++
TINEEAAEW	72	B5801	7	5	B58s	B58s	+

a Ranks<10 are considered to be high.

b All peptides listed are predicted to be transported by TAP [36] and cleaved by the proteasome [35].

c The position of each epitope is given with respect to HXB2-p24 (as in the HIV-1 immunology database).

d The HLA molecule by which the epitope is predicted to be presented.

e –indicates that there is no reliable prediction tool for this HLA type.

f The rank of the peptide among all 9-mers from HIV-1 that are predicted to bind the specific HLA molecule (where 1 denotes the best binding epitope) using the SMM method.

g The same predicted rank using a neural network based predictor (NetMHC).

h The HLA supertype to which the HLA molecule belongs according to Sette *et al.* [38].

i The HLA supertype to which the HLA molecule belongs according to Lund *et al.* [59]).

j Peptides that have been experimentally confirmed to bind the two-digit HLA type are denoted by +, the four-digit HLA allele by ++, and by HLA molecules from the same supertype as the predicted HLA type by dots.

### Why is preferential targeting of p24 protective?

Why would preferential targeting of the p24 protein provide better protection against HIV disease progression than immune responses to other HIV-1 proteins? A likely contributor is the fact that the p24 capsid protein is one of the most functionally and structurally constrained proteins of HIV-1 [3], [26], [39]. It has been shown that point mutations in the capsid surface markedly reduce viral fitness [3], [40], [41]. In contrast to such constraints on p24, the Nef protein is known to be polymorphic [26]. During acute infection, immune responses to Nef are typically replaced by responses to more conserved regions of HIV-1 [42]. The level of protection conferred by different HLA molecules may correlate with the loss of viral fitness resulting from CTL escape; a large viral fitness cost may either lead to less frequent CTL escape or to viral attenuation upon CTL escape.

**Figure 4:**
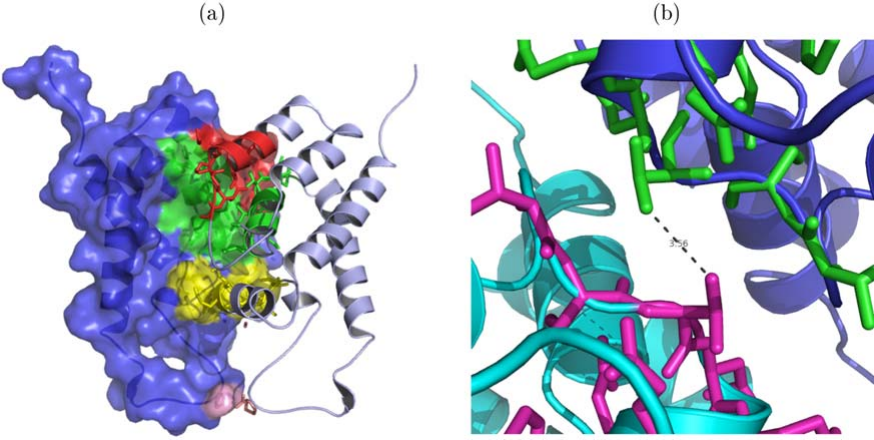
Crystal structure of a p24 dimer. Entry 1AFV from the protein data bank is shown (PDB, www.rcsb.org/pdb) [44], [45]. (A) One monomer is shown in combined cartoon and surface representation to show the extent of the surface exposed part of the epitopes. For clarity, the other monomer is shown only in cartoon representation. The epitopes located in the dimer interface are highlighted in red (IW9), yellow (TW10) and green (DV9), and are also shown in stick representation. (B) A close-up of the dimer interface. The two monomers are shown in dark blue and cyan, and the TW10 epitopes are shown in green and magenta stick representations. The hydrophobic contact between T110 from one monomer to T110 in the other monomer is indicated by a dashed line.

The epitopes targeted by protective HLA alleles indeed lie in relatively constrained regions of the p24 protein. The epitope TSTLQEQIGW (TW10, p24 positions 108 to 117) was found to trigger an immunodominant CTL response in individuals expressing HLA-B57 or B58 [9]. Mutations in this epitope allow for CTL escape, but are associated with a large viral fitness cost. For example, the Thr→Asn mutation at p24–110 causes a 10-fold reduction in the replication rate of the virus [3]. Similarly, Ala→Glu escape in another HLA-B57 immunodominant epitope, KAFSPEVIPMF (KF11, p24 positions 30 to 40), severely reduces the viral replicative capacity [43]. In the crystal structure of p24 [44], the N-terminal domain can be seen to form a homo-dimer [45], which is known to be important in Gag assembly [46]–[48]. The two monomers have contact at three extended regions (see Figure 4A), two of which have been shown to contain CTL epitopes that induce protective responses in HLA-B57 or B58-positive individuals (i.e. TW10 and ISPRTLNAW, IW9, p24 positions 15-23 [5]). The third region at the dimer interface (QDLNMMLNIVGG, p24 positions 50-61) contains an epitope restricted by HLA-B14 (DLNMMLNIV, DV9) [49], which is also associated with a low relative hazard of HIV disease progression (RH = 0.7 [13]). Recently, Martinez-Picado *et al.* [50] proposed that the reduced fitness of the T110N mutant is due to the loss of the hydrogen bond between the side chain of the hydroxyl group of T110 and the backbone amide of E113, which would destabilize helix 6 of the N-terminal domain of p24. Analysis of the dimeric structure of p24 shows that T110 also has a very central position at the protein-protein interface, being one of three residues that are in contact with their corresponding residue in the other monomer (see Figure 4B). If the threonine residue is replaced by an asparagine, the symmetric hydrophobic contact is replaced by a polar contact, which is expected to disturb the dimeric interface, and thereby to destabilize the HIV capsid. In combination with the intramolecular destabilizing effect on helix 6, this destabilization of the HIV capsid may explain why CTL escape mutations at these sites are associated with a large viral fitness cost.

## Discussion

Our results demonstrate that the association between HLA molecules conferring slow HIV-1 disease progression and CTL responses to HIV-1 Gag is due to an intrinsic preference of these HLA alleles for peptides from the p24 protein, and not merely a side-effect of the high immunogenicity of the Gag protein. Several recent studies have also established a link between low HIV viral loads and CTL responses against Gag or p24 [7], [18]–[24]. Bailey *et al.* [7] reported that CTL responses in elite suppressors of HIV infection focus on HLA-B57 restricted Gag epitopes. Moreover, a significant negative correlation between the magnitude of the CD8 T cell response and HIV-1 viral load was only found for responses against p24 and not for responses against other HIV-1 proteins [20], [22], [23]. When we performed a partial correlation on data published by Frahm *et al.* [26], who determined CTL responses to a large panel of HIV-1 peptides spanning all HIV-1 proteins in 150 infected individuals, we found that the number of CTL responses directed against p24 correlated positively with the CD4^+^ T cell count (*p* = 0.04) and negatively with viral load (*p* = 0.01), while the number of CTL responses against Nef correlated *positively* with viral load (*p* = 0.03) and not with the CD4^+^ T cell count (*p* = 0.96) (unpublished results). Interestingly, the main HIV-1 specific CTL responses in chimpanzees, which have a low viral load and do not develop AIDS, have been found to be directed against the very same peptides from p24 that are targeted by human individuals with HLA-B57 or B27, who tend to be long-term non-progressors [1], [51]. All these data suggest that preferential targeting of p24 delays disease progression. However, previous studies could never rule out the possibility that the apparent preference for p24 was a side-effect of the high immunogenicity of p24, causing immune responses to p24 to be better maintained than other responses in individuals with low viral load. Our analyses based on HLA-peptide binding predictions conclusively show that HLA alleles associated with slow HIV disease progression have an intrinsic preference for peptides from the HIV p24 protein. Our results thereby suggest that the class I restricted CTL immune response, particularly against p24, plays a key role in controlling HIV-1 infection.

Why then is preferential targeting of p24 beneficial? The first reason that comes to mind is that p24 is one of the most functionally and structurally constrained proteins of HIV. P24 contains a stretch of 20 amino acids which is conserved across retroviruses, and is essential for viral assembly, maturation, and infectivity [52]. This region contains a B14 epitope (a low RH allele), and Wagner *et al.* [53] found that all mutations abrogating the CTL response to this epitope drastically reduced the replication capacity of the virus. In line with this, we found several HIV epitopes that are presented by protective HLA molecules to be located at the dimer interface of p24, which is expected to be sensitive to mutations. The peptide binding motifs of HLA alleles with low and high relative hazards (Figure 3) also suggest that low RH alleles tend to present peptides that are more sensitive to mutations, because they prefer tryptophan (W) at position 9. Tryptophan is the only amino acid coded by a single triplet. Any mutation in a tryptophan triplet will thus lead to an amino acid substitution or a stop-codon. Due to the unusual side chain properties of tryptophan, such amino acid substitutions tend to affect the protein structure and function. As a consequence, tryptophan is the most conserved amino acid. HLA alleles with a high RH, on the other hand, prefer proline (P) at position 2, which is coded by 4 triplets, and is known to be less conserved than tryptophan. HLA alleles with a low RH thus seem to preferentially bind the parts of the HIV genome that are most sensitive to mutations. This may in part explain why they confer better protection against disease progression.

Since HIV-1 proteins other than p24 may also contain functionally and structurally constrained regions, we investigated if there is a general correlation between the relative hazard of HLA alleles and the tendency to target constrained parts of the HIV-1 proteome. We predicted the HIV-1 epitopes for a large number of HLA alleles and used the entropy of each predicted epitope as a measure of functional and structural constraint. The Shannon entropy [32] of each residue in the HIV-1 proteome was calculated using the HIV-1 protein alignments for clade B available in the Los Alamos HIV database (September 2005). Surprisingly, the average entropies of the three predicted best-binding epitopes for HLA alleles with a low RH were not significantly different from the ones presented by HLA alleles with a high RH (Mann-Whitney, *p* = 0.87, unpublished results). There was also no significant correlation between the entropy score of the best-binding predicted epitopes and the RH of the HLA presenting the epitope (*p* = 0.79, unpublished results). Presentation of conserved HIV epitopes is thus not explaining the difference between HLA alleles associated with slow and rapid disease progression.

There are two non-mutually exclusive explanations for this surprising finding. The first is that targeting conserved epitopes is required but not sufficient to delay disease progression (see also [23]). Apart from its conservedness, p24 may have other properties that explain why CTL responses against p24 are most beneficial. Possible factors include the fact that p24 is one of the most immunogenic and abundant HIV proteins. An immature HIV particle contains approximately 1500 copies of p24, while other HIV proteins are present at much lower copy numbers [54]. P24 epitopes are therefore expected to induce stronger immune responses than other HIV epitopes. Additionally, it was recently shown that p24 can be detected within two hours after the infection of a target cell, which is well before other HIV-1 proteins are produced, and before Nef can down-regulate HLA expression [55]. The origin of the early expressed p24 is probably the large amount of p24 packaged in viral particles [54]. Preferential targeting of conserved epitopes from an early and abundantly expressed, highly immunogenic protein, may hence be the clue to slowing down disease progression.

A second explanation is that entropy is not the correct measure of functional and structural constraints. Indeed, peptides that are hardly constrained may nevertheless have a low entropy if there is no strong CTL pressure on the peptide, or if the HLA molecule by which the peptide is restricted is very rare in the human population. Conversely, peptides may have a high entropy despite functional or structural constraint if the CTL pressure on the peptide is so high that the peptide mutates despite a high viral fitness cost [56]. The latter is exactly what has been described for p24 epitopes targeted by elite suppressors of HIV-1 [7]. Despite the lack of correlation between the relative hazard of HIV disease progression and the tendency to present peptides with low entropy, preferential targeting of functionally and structurally constrained regions of HIV-1 may thus be key to slowing down disease progression.

HLA-B27 was the first HLA that was described to be associated with slow HIV-1 disease progression [57]. Our analyses reveal an important difference between HLA-B27 on the one hand and HLA-B57 and B58 on the other. While the latter two HLA alleles target at least 3 different p24 peptides with high affinity, HLA-B27 has only one good-binding p24 peptide (see Figure 1). It has been reported that CTL escape of this epitope caused a rapid increase of viremia in an HLA-B27-positive long-term non-progressing child [58]. Our analyses suggest that this abrupt breakage of protection may be caused by the absence of other protective anti-p24 CTL responses.

The differences between HLA alleles associated with slow and rapid disease progression have also been sought in their frequencies in the human population. Trachtenberg *et al.* [15] demonstrated a significant correlation between the population frequency of HLA supertypes [38], [59] and the HIV-1 viral load at set point in a large group of HIV-1 infected homosexual men. Scherer *et al.* [16] extended these results by showing that common HLA alleles are associated with a lack of CTL responses to known HIV-1 epitopes, further supporting the idea that HIV-1 is adapting to the most common HLA molecules in the human population. Frahm *et al.* [17] demonstrated that an HLA molecule associated with a low viral load in a population in which the HLA is rare, lacked this association in another population where the HLA is common. Data are conflicting, however, because no significant correlation could be found between the relative hazard of HIV disease progression and the population frequency of HLA molecules [13].

Whatever the effect of the population frequency of HLA molecules on the rate of HIV disease progression is, the current study shows that qualitative differences in the epitopes targeted by different HLA molecules contribute to the association between HLA molecules and the rate of HIV-1 disease progression.

## References

[1] Carrington M, O'Brien SJ (2003). The influence of HLA genotype on AIDS.. Annu Rev Med.

[2] Moore CB, John M, James IR, Christiansen FT, Witt CS (2002). Evidence of HIV-1 adaptation to HLA-restricted immune responses at a population level.. Science.

[3] Leslie AJ, Pfafferott KJ, Chetty P, Draenert R, Addo MM (2004). HIV evolution: CTL escape mutation and reversion after transmission.. Nat Med.

[4] Friedrich TC, Dodds EJ, Yant LJ, Vojnov L, Rudersdorf R (2004). Reversion of CTL escape-variant immunodeficiency viruses in vivo.. Nat Med.

[5] Klein MR, Van der Burg SH, Hovenkamp E, Holwerda AM, Drijfhout JW (1998). Characterization of HLA-B57-restricted human immunodeficiency virus type 1 Gag- and RT-specific cytotoxic T lymphocyte responses.. J Gen Virol.

[6] Gillespie GM, Kaul R, Dong T, Yang HB, Rostron T (2002). Cross-reactive cytotoxic T lymphocytes against a HIV-1 p24 epitope in slow progressors with B*57.. AIDS.

[7] Bailey JR, Williams TM, Siliciano RF, Blankson JN (2006). Maintenance of viral suppression in HIV-1-infected HLA-B*57^+^ elite suppressors despite CTL escape mutations.. J Exp Med.

[8] Jansen CA, Kostense S, Vandenberghe K, Nanlohy NM, De Cuyper IM (2005). High responsiveness of HLA-B57-restricted Gag-specific CD8^+^ T cells in vitro may contribute to the protective effect of HLA-B57 in HIV-infection.. Eur J Immunol.

[9] Altfeld M, Addo MM, Rosenberg ES, Hecht FM, Lee PK (2003). Influence of HLA-B57 on clinical presentation and viral control during acute HIV-1 infection.. AIDS.

[10] Altfeld M, Kalife ET, Qi Y, Streeck H, Lichterfeld M (2006). HLA alleles associated with delayed progression to AIDS contribute strongly to the initial CD8^+^ T cell response against HIV-1.. PLoS Med.

[11] Frahm N, Adams S, Kiepiela P, Linde CH, Hewitt HS (2005). HLA-B63 presents HLA-B57/B58-restricted cytotoxic T-lymphocyte epitopes and is associated with low human immunodeficiency virus load.. J Virol.

[12] Goulder PJ, Edwards A, Phillips RE, McMichael AJ (1997). Identification of a novel HLA-B*2705-restricted cytotoxic T-lymphocyte epitope within a conserved region of HIV-1 Nef.. AIDS.

[13] Gao X, Nelson GW, Karacki P, Martin MP, Phair J (2001). Effect of a single amino acid change in MHC class I molecules on the rate of progression to AIDS.. N Engl J Med.

[14] Carrington M, Nelson GW, Martin MP, Kissner T, Vlahov D (1999). HLA and HIV-1: heterozygote advantage and B*35-Cw*04 disadvantage.. Science.

[15] Trachtenberg E, Korber B, Sollars C, Kepler TB, Hraber PT (2003). Advantage of rare HLA supertype in HIV disease progression.. Nat Med.

[16] Scherer A, Frater J, Oxenius A, Agudelo J, Price DA (2004). Quantifiable cytotoxic T lymphocyte responses and HLA-related risk of progression to AIDS.. Proc Natl Acad Sci USA.

[17] Frahm N, Kiepiela P, Adams S, Linde CH, Hewitt HS (2006). Control of human immunodeficiency virus replication by cytotoxic T lymphocytes targeting subdominant epitopes.. Nat Immunol.

[18] Buseyne F, Le Chenadec J, Corre B, Porrot F, Burgard M (2002). Inverse correlation between memory Gag-specific cytotoxic T lymphocytes and viral replication in human immunodeficiency virus-infected children.. J Infect Dis.

[19] Edwards BH, Bansal A, Sabbaj S, Bakari J, Mulligan MJ (2002). Magnitude of functional CD8^+^ T-cell responses to the gag protein of human immunodeficiency virus type 1 correlates inversely with viral load in plasma.. J Virol.

[20] Novitsky V, Gilbert P, Peter T, McLane MF, Gaolekwe S (2003). Association between virus-specific T-cell responses and plasma viral load in human immunodeficiency virus type 1 subtype C infection.. J Virol.

[21] Masemola A, Mashishi T, Khoury G, Mohube P, Mokgotho P (2004). Hierarchical targeting of subtype C human immunodeficiency virus type 1 proteins by CD8^+^ T cells: correlation with viral load.. J Virol.

[22] Zuniga R, Lucchetti A, Galvan P, Sanchez S, Sanchez C (2006). Relative dominance of Gag p24-specific cytotoxic T lymphocytes is associated with human immunodeficiency virus control.. J Virol.

[23] Kiepiela P, Ngumbela K, Thobakgale C, Ramduth D, Honeyborne I (2007). CD8^+^ T-cell responses to different HIV proteins have discordant associations with viral load.. Nat Med.

[24] Honeyborne I, Prendergast A, Pereyra F, Leslie A, Crawford H (2007). Control of human immunodeficiency virus type 1 is associated with HLA-B*13 and targeting of multiple gag-specific CD8^+^ T-cell epitopes.. J Virol.

[25] Streeck H, Lichterfeld M, Alter G, Meier A, Teigen N (2007). Recognition of a defined region within p24 Gag by CD8^+^ T cells during primary human immunodeficiency virus type 1 infection in individuals expressing protective HLA class I alleles.. J Virol.

[26] Frahm N, Korber BT, Adams CM, Szinger JJ, Draenert R (2004). Consistent cytotoxic T-lymphocyte targeting of immunodominant regions in human immunodeficiency virus across multiple ethnicities.. J Virol.

[27] Peters B, Sette A (2005). Generating quantitative models describing the sequence specificity of biological processes with the stabilized matrix method.. BMC Bioinformatics.

[28] Buus S, Lauemoller SL, Worning P, Keşmir C, Frimurer T (2003). Sensitive quantitative predictions of peptide-MHC binding by a ‘Query by Committee’ artificial neural network approach.. Tissue Antigens.

[29] Nielsen M, Lundegaard C, Worning P, Lauemoller SL, Lamberth K (2003). Reliable prediction of T-cell epitopes using neural networks with novel sequence representations.. Protein Sci.

[30] Sturniolo T, Bono E, Ding J, Raddrizzani L, Tuereci O (1999). Generation of tissue-specific and promiscuous HLA ligand databases using DNA microarrays and virtual HLA class II matrices.. Nat Biotechnol.

[31] Schneider TD, Stephens RM (1990). Sequence logos: a new way to display consensus sequences.. Nucleic Acids Res.

[32] Shannon CE (1948). A mathematical theory of communication.. Bell System Tech J.

[33] Simes RJ (1986). An improved Bonferroni procedure for multiple tests of significance.. Biometrika.

[34] Assarsson E, Sidney J, Oseroff C, Pasquetto V, Bui HH (2007). A quantitative analysis of the variables affecting the repertoire of T cell specificities recognized after vaccinia virus infection.. J Immunol.

[35] Keşmir C, Nussbaum AK, Schild H, Detours V, Brunak S (2002). Prediction of proteasome cleavage motifs by neural networks.. Protein Eng.

[36] Peters B, Bulik S, Tampe R, Van Endert PM, Holzhutter HG (2003). Identifying MHC class I epitopes by predicting the TAP transport efficiency of epitope precursors.. J Immunol.

[37] Korber B, Muldoon M, Theiler J, Gao F, Gupta R (2000). Timing the ancestor of the HIV-1 pandemic strains.. Science.

[38] Sette A, Sidney J (1999). Nine major HLA class I supertypes account for the vast preponderance of HLA-A and -B polymorphism.. Immunogenetics.

[39] Novitsky V, Smith UR, Gilbert P, McLane MF, Chigwedere P (2002). Human immunodeficiency virus type 1 subtype C molecular phylogeny: consensus sequence for an AIDS vaccine design?. J Virol.

[40] Von Schwedler UK, Stray KM, Garrus JE, Sundquist WI (2003). Functional surfaces of the human immunodeficiency virus type 1 capsid protein.. J Virol.

[41] Peyerl FW, Bazick HS, Newberg MH, Barouch DH, Sodroski J (2004). Fitness costs limit viral escape from cytotoxic T lymphocytes at a structurally constrained epitope.. J Virol.

[42] Lichterfeld M, Yu XG, Cohen D, Addo MM, Malenfant J (2004). HIV-1 Nef is preferentially recognized by CD8 T cells in primary HIV-1 infection despite a relatively high degree of genetic diversity.. AIDS.

[43] Crawford H, Prado JG, Leslie A, Hue S, Honeyborne I (2007). Compensatory mutation partially restores fitness and delays reversion of escape mutation within the immunodominant HLA-B*5703-restricted Gag epitope in chronic human immunodeficiency virus type 1 infection.. J Virol.

[44] Berman HM, Westbrook J, Feng Z, Gilliland G, Bhat TN (2000). The Protein Data Bank.. Nucleic Acids Res.

[45] Momany C, Kovari LC, Prongay AJ, Keller W, Gitti RK (1996). Crystal structure of dimeric HIV-1 capsid protein.. Nat Struct Biol.

[46] Accola MA, Strack B, Gottlinger HG (2000). Efficient particle production by minimal Gag constructs which retain the carboxy-terminal domain of human immunodeficiency virus type 1 capsid-p2 and a late assembly domain.. J Virol.

[47] Johnson MC, Scobie HM, Ma YM, Vogt VM (2002). Nucleic acid-independent retrovirus assembly can be driven by dimerization.. J Virol.

[48] Zhang Y, Qian H, Love Z, Barklis E (1998). Analysis of the assembly function of the human immunodeficiency virus type 1 gag protein nucleocapsid domain.. J Virol.

[49] Kaul R, Plummer FA, Kimani J, Dong T, Kiama P (2000). HIV-1-specific mucosal CD8^+^ lymphocyte responses in the cervix of HIV-1-resistant prostitutes in Nairobi.. J Immunol.

[50] Martinez-Picado J, Prado JG, Fry EE, Pfafferott K, Leslie A (2006). Fitness cost of escape mutations in p24 Gag in association with control of human immunodeficiency virus type 1.. J Virol.

[51] Balla-Jhagjhoorsingh SS, Koopman G, Mooij P, Haaksma TG, Teeuwsen VJ (1999). Conserved CTL epitopes shared between HIV-infected human long-term survivors and chimpanzees.. J Immunol.

[52] Gamble TR, Yoo S, Vajdos FF, Von Schwedler UK, Worthylake DK (1997). Structure of the carboxyl-terminal dimerization domain of the HIV-1 capsid protein.. Science.

[53] Wagner R, Leschonsky B, Harrer E, Paulus C, Weber C (1999). Molecular and functional analysis of a conserved CTL epitope in HIV-1 p24 recognized from a long-term nonprogressor: constraints on immune escape associated with targeting a sequence essential for viral replication.. J Immunol.

[54] Briggs JA, Simon MN, Gross I, Krausslich HG, Fuller SD (2004). The stoichiometry of Gag protein in HIV-1.. Nat Struct Mol Biol.

[55] Sacha JB, Chung C, Rakasz EG, Spencer SP, Jonas AK (2007). Gag-specific CD8^+^ T lymphocytes recognize infected cells before AIDS-virus integration and viral protein expression.. J Immunol.

[56] Frater AJ, Brown H, Oxenius A, Gunthard HF, Hirschel B (2007). Effective T-cell responses select human immunodeficiency virus mutants and slow disease progression.. J Virol.

[57] Nixon DF, Townsend AR, Elvin JG, Rizza CR, Gallwey J (1988). HIV-1 gag-specific cytotoxic T lymphocytes defined with recombinant vaccinia virus and synthetic peptides.. Nature.

[58] Feeney ME, Tang Y, Roosevelt KA, Leslie AJ, McIntosh K (2004). Immune escape precedes breakthrough human immunodeficiency virus type 1 viremia and broadening of the cytotoxic T-lymphocyte response in an HLA-B27-positive long-term-nonprogressing child.. J Virol.

[59] Lund O, Nielsen M, Keşmir C, Petersen AG, Lundegaard C (2004). Definition of supertypes for HLA molecules using clustering of specificity matrices.. Immunogenetics.

